# Utility of genetic risk scores in type 1 diabetes

**DOI:** 10.1007/s00125-023-05955-y

**Published:** 2023-07-13

**Authors:** Amber M. Luckett, Michael N. Weedon, Gareth Hawkes, R. David Leslie, Richard A. Oram, Struan F. A. Grant

**Affiliations:** 1 University of Exeter College of Medicine and Health, Exeter, UK; 2 Blizard Institute, Queen Mary University of London, London, UK; 3 Royal Devon University Healthcare NHS Foundation Trust, Exeter, UK; 4 Division of Human Genetics, Children’s Hospital of Philadelphia, Philadelphia, PA USA; 5 Division of Diabetes and Endocrinology, Children’s Hospital of Philadelphia, Philadelphia, PA USA; 6 Center for Spatial and Functional Genomics, Children’s Hospital of Philadelphia, Philadelphia, PA USA; 7 Department of Genetics, Perelman School of Medicine, University of Pennsylvania, Philadelphia, PA USA; 8 Institute for Diabetes, Obesity and Metabolism, Perelman School of Medicine, University of Pennsylvania, Philadelphia, PA USA; 9 Department of Pediatrics, Perelman School of Medicine, University of Pennsylvania, Philadelphia, PA USA

**Keywords:** Autoimmune disorders, Diabetes, Genetic risk score, Genetics, Review, Type 1 diabetes

## Abstract

Iterative advances in understanding of the genetics of type 1 diabetes have identified >70 genetic regions associated with risk of the disease, including strong associations across the HLA class II region that account for >50% of heritability. The increased availability of genetic data combined with the decreased costs of generating these data, have facilitated the development of polygenic scores that aggregate risk variants from associated loci into a single number: either a genetic risk score (GRS) or a polygenic risk score (PRS). PRSs incorporate the risk of many possibly correlated variants from across the genome, even if they do not reach genome-wide significance, whereas GRSs estimate the cumulative contribution of a smaller subset of genetic variants that reach genome-wide significance. Type 1 diabetes GRSs have utility in diabetes classification, aiding discrimination between type 1 diabetes, type 2 diabetes and MODY. Type 1 diabetes GRSs are also being used in newborn screening studies to identify infants at risk of future presentation of the disease. Most early studies of type 1 diabetes genetics have been conducted in European ancestry populations, but, to develop accurate GRSs across diverse ancestries, large case–control cohorts from non-European populations are still needed. The current barriers to GRS implementation within healthcare are mainly related to a lack of guidance and knowledge on integration with other biomarkers and clinical variables. Once these limitations are addressed, there is huge potential for ‘test and treat’ approaches to be used to tailor care for individuals with type 1 diabetes.

**Graphical Abstract:**

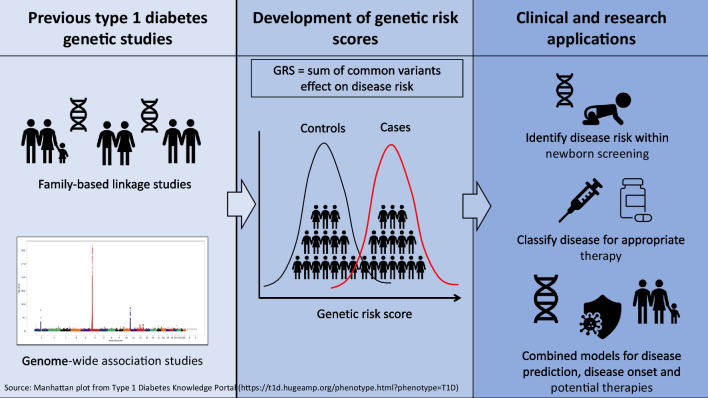

**Supplementary Information:**

The online version contains a slideset of the figures for download available at 10.1007/s00125-023-05955-y.

## Introduction

The pathogenesis of type 1 diabetes is commonly described as occurring in stages [1, 2]. Background genetic risk combined with environmental triggers are thought to contribute to the initiation of autoimmunity, commonly defined by the presence of islet-specific autoantibodies to insulin, IA-2, GAD and ZnT8. The stages are defined as follows: stage 1, presence of two or more islet-specific autoantibodies [3]; stage 2, progression to dysglycaemia; and stage 3, meeting standard clinical diagnostic criteria for diabetes [4, 5].

Type 1 diabetes is a common complex disease with numerous associated loci across the genome and particularly strong HLA associations [6–10]. Genetic predisposition is an important contributor to type 1 diabetes development risk. Historically, genetic risk was assessed by family history or measured by HLA typing and genotyping of other type 1 diabetes-associated loci [6, 8–13]. Recently, summarising genetic risk for common diseases as genetic risk scores (GRSs) and polygenic risk scores (PRSs) has proved an efficient method to measure heritable risk [14, 15].

Here, we describe the genetic architecture of type 1 diabetes, focusing on GRS development and the utility of GRSs for the classification and prediction of type 1 diabetes and their potential for integration into clinical care.

## Heritability of type 1 diabetes

Twin and family studies provided evidence for a substantial heritable component of type 1 diabetes, which declines substantially with increasing age at diagnosis [6, 8–13, 16–18]. Concordance rates within monozygotic and dizygotic twin pairs suggest a risk of >50% and ~8%, respectively [9–13, 16–18]. Sibling concordance rates range from 6% to 10%, with a risk of 6–9% for offspring of an affected father and 1–4% for offspring of an affected mother, suggesting relative maternal protection from type 1 diabetes [9–13, 16–18].

Family-based linkage analyses attributed a large proportion of type 1 diabetes heritability to variation in the class II HLA genes residing within the MHC region on chromosome 6 [19]. The HLA haplotypes (combination of alleles at multiple loci on the same chromosome) DRB1*03:01–DQA1*05:01–DQB1*02:01 (*DR3*) and DRB1*04:XX–DQA1*03:01–DQB1*03:02 (*DR4-DQ8*) confer the highest type 1 diabetes genetic risk and are relatively common in European ancestry populations (Table 1) [6–8, 13, 20, 21]. Each person has two haplotypes, which in combination can be referred to as an HLA diplotype [22]. A single copy of a *DR3* or *DR4-DQ8* haplotype increases the odds of type 1 diabetes by 4.5 and 7, respectively [6]. *DR3*/*DR4-DQ8* heterozygosity increases the type 1 diabetes risk by over 30-fold, a substantially higher risk than in the case of homozygosity for either haplotype. Conversely, some HLA haplotypes, such as DRB1*15:01–DQA1*01:02–DQB1*06:02 (*DR15-DQ6.2*), are associated with strong and sometimes dominant reductions in type 1 diabetes risk [6, 20]. The degree of risk and protection conferred by HLA haplotypes varies with age, for example the impact of class I HLA alleles declines in individuals diagnosed after age 7 years [13, 23].

**Table 1:** Prevalence of five common type 1 diabetes risk or protective *HLA-DR-DQ* haplotypes in different ethnic groups [13]

Haplotype	European populations (%)	Middle Eastern populations (%)	African populations (%)	East Asian populations (%)	South Asian populations (%)
*DR3-DQ2.5* (DRB1*03:01–DQA1*05:01–DQB1*02:01)	12.2	8.9	7.2	6.8	7.5
*DR4-DQ8.1* (DRB1*04:XX–DQA1*03:01–DQB1*03:02)	9.8	8.3	4.7	5.7	8.9
*DR9-DQ9.3* (DRB1*09:01-DQA1*03:02-DQB1*03:03)	1.0	0.4	0.0	15.5	0.6
*DR15-DQ6.1* (DRB1*15:02-DQA1*01:03-DQB1*06:01) and *DR15-DQ6.2* (DRB1*15:01-DQA1*01:02-DQB1*06:02)	14.1	9.1	12.8	10.1	19.1
*DR15-DQ6.3* (DRB1*15:XX-DQA1*01:03-DQB1*06:03) and *DR15-DQ6.9* (DRB1*15:XX-DQA1*01:02-DQB1*06:09)	6.8	5.3	5.9	2.7	8.3

**Fig. 1:**
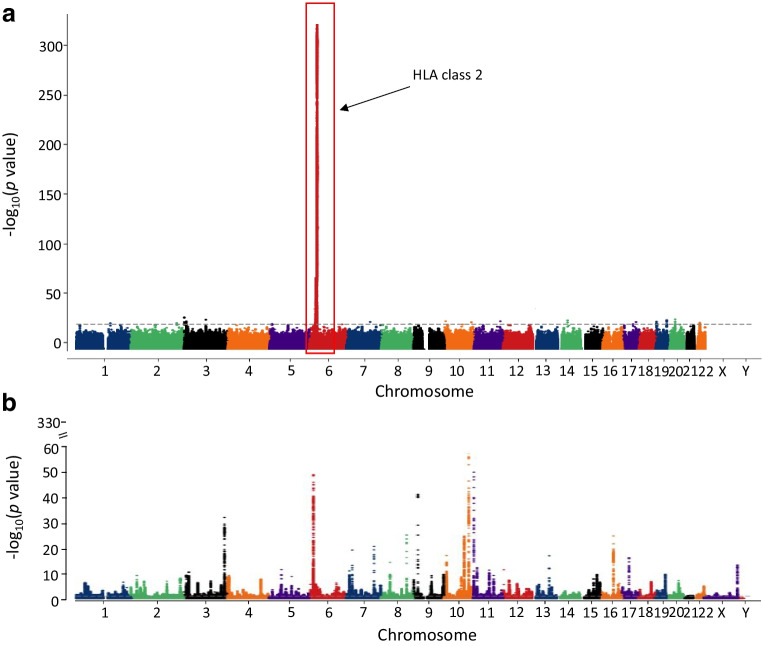
Manhattan plots of genotyped and imputed genetic variants associated with type 1 and type 2 diabetes across the genome from the T1D Knowledge Portal [30, 31]. (**a**) Manhattan plot of type 1 diabetes variants, showing the most dominant association present in the HLA region on chromosome 6 (red box). Common risk variants that have been identified include loci that harbour the insulin (*INS*), cytotoxic T-lymphocyte associated protein 4 (*CTLA4*), protein tyrosine phosphatase non-receptor type 22 (*PTPN22*) and interferon induced with helicase C domain 1 (*IFIH1* [also known as *MDA-5*]) genes and the regions around the IL-2 receptor alpha gene (*IL2RA* [also known as *CD25*]) [28–32]. (**b**) Manhattan plot identifying multiple common type 2 diabetes variants, each with a similar moderate effect. Each point represents a genetic variant. This figure is available as part of a downloadable slideset

**Fig. 2:**
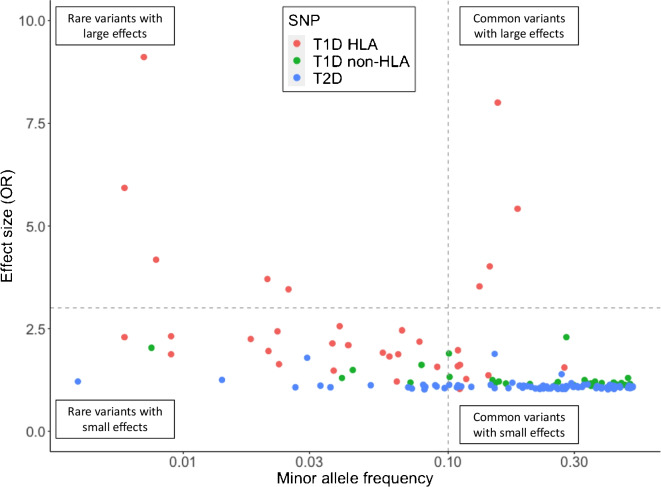
Type 1 diabetes genetic risk includes common HLA variants with large effects. The 35 type 1 diabetes-associated HLA SNPs, 32 type 1 diabetes-associated non-HLA SNPs and 89 type 2 diabetes-associated SNPs highlight the large impact of HLA variants in type 1 diabetes, compared with the small effect of common type 2 diabetes variants. Minor allele frequencies were ascertained from the UK Biobank European American subset [28] and the Genome Aggregation Database (gnomAD) browser European (non-Finnish) subset [82]. Type 1 diabetes SNP effect sizes were obtained from [6]. Type 2 diabetes SNP effect sizes were obtained from [34–41, 43]. T1D, type 1 diabetes; T2D, type 2 diabetes. This figure is available as part of a downloadable slideset

Family-based linkage studies and subsequent case–control genome-wide association studies (GWAS) have identified >70 common non-HLA type 1 diabetes risk loci (Fig. 1a) [24–31]. Non-HLA risk alleles have lower effect sizes (typical ORs ~0.5–2.3) than the strongest HLA risk allele, with only the insulin (*INS*) variable number of tandem repeats (VNTR) risk-increasing allele having an OR in excess of 2 [6]. The >50% of heritable risk explained by the HLA region is similar to what is seen in other autoimmune diseases but contrasts with the situation in other common complex diseases, for example type 2 diabetes, in which heritability is more equally distributed across all chromosomes (as shown in Figs 1b and 2) [6, 25, 28–43]. Identifying variants with small effect sizes, which can combine to have an additive effect on disease risk, is necessary for capturing the full repertoire of genetic risk to aid in unravelling the biology driving pathogenesis [6, 7]. Despite most genetic studies focusing on childhood-onset disease, new cases of type 1 diabetes occur throughout adulthood [44, 45]. There is a clear need for further large association studies of adult-onset type 1 diabetes to assess the genetic contribution to age at diagnosis and clinical heterogeneity of the disease.

Measurement of individual variants has not been useful for individual risk prediction or disease classification [8]; instead, PRSs and GRSs aggregate the contribution of associated loci to disease risk [14, 15]. PRSs use a probabilistic approach to incorporate the risk of many possibly correlated variants from across the genome even if they do not reach genome-wide significance, whereas GRSs estimate the cumulative contribution of genetic variants that are significantly associated with disease in GWAS [14, 15]. The aggregation of genetic risk has utility in mechanistic studies (e.g. Mendelian randomisation) as well as practical clinical applications (as discussed later in this review).

## Development of type 1 diabetes GRSs

**Fig. 3:**
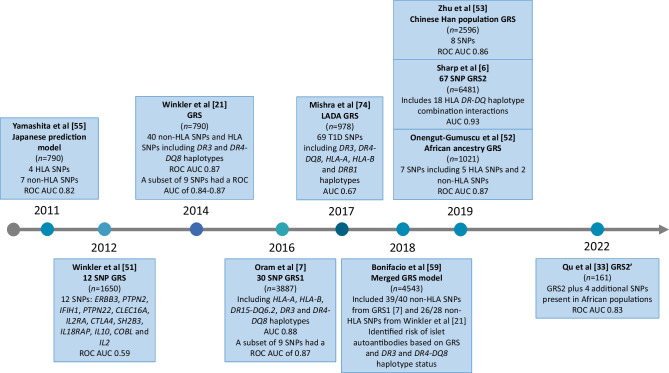
Timeline of type 1 diabetes GRS development. ROC, receiver operating characteristic.This figure is available as part of a downloadable slideset

Natural history studies have provided insight into type 1 diabetes progression in individuals at elevated genetic risk. Early studies, for example BABYDIAB, Diabetes Autoimmunity Study in the Young (DAISY) and the observational arm of the Type 1 Diabetes TrialNet Pathway to Prevention [46–48], used family history to recruit participants. Other studies recruited individuals based on high HLA risk (e.g. Type 1 Diabetes Prediction and Prevention [DIPP], DAISY, The Environmental Determinants of Diabetes in the Young [TEDDY] [48–50]). As increasing numbers of non-HLA variants continued to be identified in association with type 1 diabetes, this information was incorporated into disease prediction scores and rapidly tested (Fig. 3) [6, 7, 21, 33, 51–55]. A combined GRS including seven non-HLA variants and four HLA haplotypes was discriminative of type 1 diabetes, with an AUC of the receiver operating characteristic curve of 0.817 in 953 controls and 790 cases, but was not externally validated [14, 55, 56]. Winkler et al found that a GRS including 12 non-HLA loci performs modestly well for identifying type 1 diabetes risk (AUC=0.588) [51]. They then showed that a type 1 diabetes GRS incorporating 40 non-HLA variants and typing of *DR3* and *DR4-DQ8* variants predicted disease development (AUC=0.84–0.87) [21]. They performed multivariable logistic regression and a Bayesian algorithm in the Type 1 Diabetes Genetics Consortium (T1DGC) dataset to develop weights for each variant and validated these in the BABYDIAB/BABYDIET datasets [21, 46]. A GRS including *DR3/DR4-DQ8* carrier status and nine non-HLA variants had similar predictive power (AUC=0.82–0.86), highlighting the skewed heritability of type 1 diabetes based on relatively few loci and the dominant role of HLA variants in future type 1 diabetes development [21].

Oram et al developed a 30 SNP type 1 diabetes GRS (termed GRS1) using *DR3* and *DR4-DQ8* HLA weights from the Winkler et al study [21] but used SNP ‘tags’ for key HLA alleles [7, 57]. Because of extensive linkage disequilibrium within the HLA region, HLA variants can be identified without the need for full HLA typing or sequencing by conventional methods [57]. GRS1 included the dominant protective (ORs ~0.03–0.05) HLA *DR15-DQ6* haplotype, common to populations of European ancestry [7, 58]. HLA information obtained from SNP tags reduces the costs of measuring genetic risk through the use of small custom genotyping panels (or genome-wide genotyping array data) [7, 8, 57]. Non-HLA GRS1 variants had weights based on ORs from the largest published GWAS and assumed an additive risk contribution [7]. GRS1 was highly effective in discriminating type 1 from type 2 diabetes (AUC=0.87), with most discriminative power provided by the top nine SNPs (AUC=0.87). Bonifacio et al combined the Winkler et al and Oram et al GRSs [7, 21] and tested this ‘merged’ GRS in the TEDDY study [59]. This merged score required only cheap custom SNP assays and formed the basis for a future large population screening study (discussed in ‘GRS utility in population screening’).

Previous HLA association studies highlighted risk variants and HLA associations that are more prominent in non-European ancestries (e.g. HLA-DRB1*09-DQA1*03-DQB1*03:03 in East Asian populations), which were not used in early GRS models [29]. Improved SNP array density coverage and larger reference datasets enabled more accurate GWAS and HLA imputation. Sharp et al developed a type 1 diabetes GRS (termed GRS2), using multiplicative interaction terms to further capture HLA class II allele contributions (https://github.com/sethsh7/hla-prs-toolkit) [6]. GRS2 includes 35 HLA and 32 non-HLA variants, along with SNP tags for 14 HLA class II alleles and interaction terms for 18 HLA DR-DQ haplotype combinations, the latter of which had not been previously included in typical GRS models. GRS2 had an AUC of 0.93 for all cases of type 1 diabetes in the T1DGC dataset (*n*=16,086), with the highest discrimination at the youngest ages, highlighting the benefit of comprehensively capturing HLA risk and the stronger genetic associations in very young children [6].

## Differences in trans-ancestry and ancestry-specific scores

**Fig. 4:**
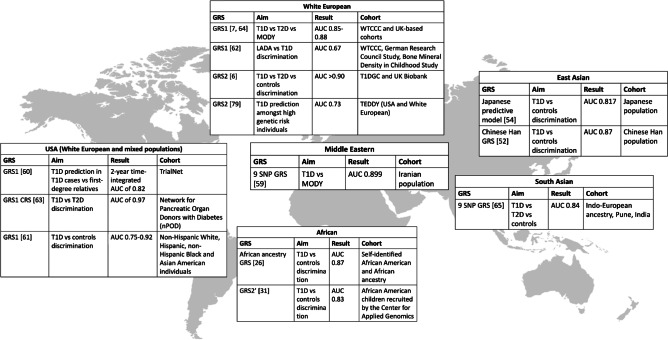
World map of GRS model performance in predicting risk of type 1 diabetes (T1D), type 2 diabetes (T2D), MODY and latent autoimmune diabetes of adults (LADA) in different cohorts and ethnic groups. WTCCC, Wellcome Trust Case Control Consortium. Image by rawpixel.com on Freepik. This figure is available as part of a downloadable slideset

The GRS models described above were derived from European ancestry cohort studies and their discriminatory power may differ in cohorts of different ancestries (Fig. 4) [6, 7, 13, 33, 53, 55, 59–66]. A nine SNP GRS was discriminative of type 1 diabetes in a South Asian population, with an AUC of 0.84, only slightly lower than that in European ancestry individuals (AUC=0.87) [66]. The lower nine SNP GRS distribution in the South Asian population than in a European cohort was partly explained by background allele frequency differences between strong-effect HLA *DR-DQ* alleles (Table 1) [66]. The strongly protective HLA *DR15-DQ6.2* haplotype is almost absent from South Asia but is common among European populations, impacting the risk prediction of GRS1 and GRS2. Furthermore, *DR3* has a greater impact on type 1 diabetes risk in South Asian populations than in European populations, whereas the association with *DR4*-*DQ8* is weaker.

Early GRSs proved discriminative in people from Europe, South Asia and Iran and in Hispanic populations, but were less discriminative in African American populations (AUC=0.75) [13, 62, 67]. Perry et al demonstrated the application of GRSs in individuals from the USA with European and Hispanic ancestry, and highlighted the need for an ancestry-specific GRS with equal discriminative power to identify type 1 diabetes in African ancestry individuals [62]. Onengut-Gumuscu et al undertook a case–control study in individuals of African ancestry and confirmed the association of HLA *DR3* and *DR4-DQ8* and six non-HLA loci with risk of type 1 diabetes. They used the top seven GWAS variants to develop a discriminative African ancestry type 1 diabetes GRS [26], which has now been validated in several cohorts. GRS2 performed similarly in Hispanic, White and Black individuals in the SEARCH for Diabetes in Youth population-based cohort; however, the score distribution differed between each ethnicity [68]. The addition of four type 1 diabetes-associated SNPs to GRS2 improved the AUC in an African American cohort and a European African cohort [33]. The discrimination of GRS2 in these instances provides further evidence that accurate inclusion of a diverse range of HLA variants, to form trans-ancestry GRS models, may improve utility across ancestries. Differences in underlying type 1 diabetes genetic risk may account for differences in GRS distribution, if HLA risk alleles are captured well. However, GRS models may need to be optimised or normalised for ancestry to achieve the best sensitivity and specificity.

In East Asian individuals, type 1 diabetes has a lower childhood prevalence and incidence. A recent GWAS of 2596 individuals with autoantibody-positive type 1 diabetes and 5082 control participants in a Chinese Han population revealed two novel type 1 diabetes risk loci and two previously reported risk loci [53]. Fine-mapping revealed a novel locus at HLA-C position 275. A GRS was developed, with a higher score associated with earlier age at type 1 diabetes diagnosis (AUC=0.87) [53]. Differences in HLA associations have also been described in Japanese populations, in which *DR3* is absent and *DR4-DQ8* is not associated with type 1 diabetes risk [55]. Typically rare in White European populations, DRB1*0405-DQB1*0401, DRB1*0802-DQB1*0302 and DRB1*0901-DQB1*0303 confer type 1 diabetes risk in Japanese populations, with DRB1*1502-DQB1*0601 being the major protective haplotype [55]. In Korean populations, the Asian-specific DRB1*0405-DQB1*0401 and DRB1*0901-DQB1*0303 haplotypes are present alongside *DR3*. The variation in HLA haplotypes based on ancestry and geographic origin suggests a need to tag these HLA alleles well, either with trans-ancestry or with ancestry-specific scores.

## GRS utility for the classification of diabetes

### Classification of type 1 and type 2 diabetes

The increasing prevalence of obesity, resulting in higher numbers of people being diagnosed with type 2 diabetes at younger ages and increased levels of obesity in people with type 1 diabetes, has made the classification of type 1 diabetes progressively more challenging [7, 45, 69]. There is also increasing recognition that type 1 diabetes can present at any age; indeed, onset occurs more frequently in adulthood than in childhood. Historically, clinical features and/or antibody status have been used to classify diabetes but the addition of information from GRSs may improve discrimination.

GRS1 was initially found to be equally discriminative of type 1 diabetes from type 2 diabetes [7] and MODY [65]. When evaluating the additional benefit of GRS1 over conventional clinical features and biomarkers, a combined model provided the best discrimination of progression to severe insulin deficiency [7]. This approach was validated in cohorts of people with adult-onset type 1 diabetes and GADA-positive type 2 diabetes, with insulin deficiency as an outcome [70, 71]. Thomas et al found that individuals with type 1 diabetes have higher GRS1 scores than individuals with type 2 diabetes (*p*<0.001) among those diagnosed over the age of 30 [69]. More recently, GRS2 was shown to be similarly discriminative of type 1 diabetes in the ancestrally diverse SEARCH for Diabetes in Youth study [68].

### Type 1 diabetes, MODY and integration into clinical care

**Fig. 5:**
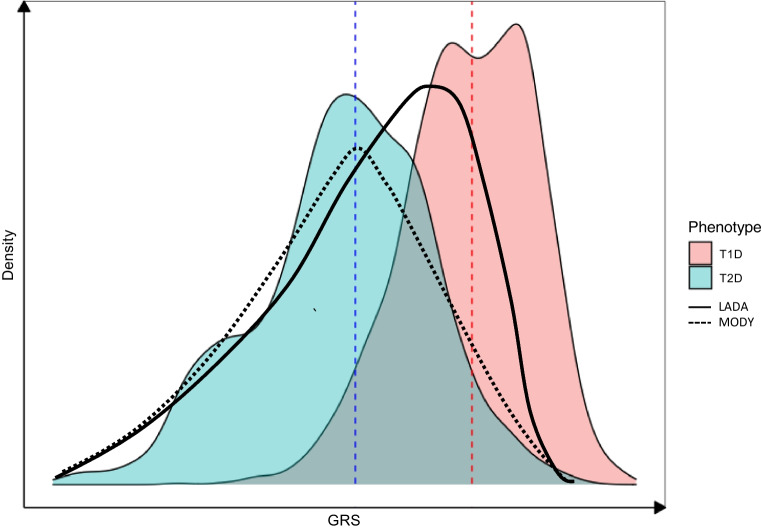
Schematic demonstrating the distribution of type 1 diabetes GRSs for type 1 diabetes, type 2 diabetes, MODY and latent autoimmune diabetes of adults (LADA) [6, 45, 54, 65]. A type 1 diabetes GRS is effective at discriminating type 1 diabetes from MODY and type 2 diabetes [65]. GRS outcomes for type 2 diabetes and MODY display similarities. GRSs for individuals with LADA overlap type 1 diabetes and type 2 diabetes GRSs [54]. The dashed blue line represents the mean GRS2 score for individuals with type 2 diabetes. The red dashed line represents the mean GRS2 score for individuals with type 1 diabetes. T1D, type 1 diabetes; T2D, type 2 diabetes. This figure is available as part of a downloadable slideset

GRSs have potential for distinguishing MODY from type 1 diabetes (Fig. 5) [65]. A high GRS1 score (>50th centile) was indicative of type 1 diabetes, while lower scores indicated an elevated probability of monogenic disease [65]. The addition of GRS1 to testing for GADA, IA2A, ZnT8A and C-peptide may help identify those likely to have monogenic diabetes.

### Utility in large cohort studies

Integration of SNP array data into large population-based datasets allows the aetiology of diabetes to be investigated without requiring additional autoantibody testing [8]. Within the UK Biobank dataset, GRS1 alone identified type 1 diabetes in 42% of adults aged 31–60 years with a high genetic risk and provided evidence that the onset of type 1 diabetes may occur at any age [45]. Individuals who were diagnosed later and those with a younger age at diagnosis had similar clinical presentations, for example diabetic ketoacidosis as a discharge diagnosis [45]. Thomas et al subsequently used a type 1 diabetes GRS to validate various approaches to clinical and healthcare record-based classification of insulin-treated diabetes where classification biomarkers are unavailable [72].

## GRSs can provide insight into the aetiology of diabetes in specific cohorts

### Early-onset diabetes

Until recently it was believed that all early-onset type 1 diabetes cases were neonatal diabetes mellitus [65, 73]. A median GRS1 cut-off in individuals with neonatal diabetes (*n*=48) with unknown genetics identified those most likely to have a monogenic form of diabetes (<50th centile) compared with probable early-onset diabetes (>50th centile) [65]. A high GRS (>95th centile) confirmed the presence of early-onset (<6 months) type 1 diabetes in 38% of infants with type 1 diabetes at <6 months [73].

### Adult-onset diabetes

Adult-onset type 1 diabetes is currently defined clinically, but may be misclassified as type 2 diabetes because of overlapping presenting clinical features and the historical view that the onset of type 1 diabetes occurs predominantly in childhood [45, 74]. In cohorts of people with adult-onset diabetes and clinically diagnosed type 2 diabetes, the presence of autoantibodies and elevated type 1 diabetes genetic risk is associated with progression to insulin treatment, highlighting a likely misdiagnosis [75]. There is a relative paucity of data on, and variability in, speed of progression to diagnosis of adult type 1 diabetes and progression to insulin deficiency [65–68, 76]. Autoantibody-positive (commonly GADA) adult cohorts defined by initial non-insulin-based therapy have previously been described as having latent autoimmune diabetes of adults (LADA), but more recently as having slowly evolving, immune-mediated diabetes of adults [73]. There is ongoing discussion as to whether this more variable phenotype (including progression to insulin dependence) represents the accepted proportion of individuals with type 2 diabetes and false-positive autoantibodies (a proportion that varies with the prior probability of type 1 diabetes), or people with overlapping type 1 diabetes and type 2 diabetes aetiologies, or people with a separate disease entity [75].

The first GWAS of LADA identified strong genetic signals associated with both type 1 and type 2 diabetes [77]. A 67 SNP type 1 diabetes GRS better predicted LADA status (AUC=0.67) than a type 2 diabetes GRS (AUC=0.57), suggesting genetic similarity between type 1 diabetes and LADA (Fig. 5) [74]. The LADA GWAS identified a novel independent signal in the *PFKFB3* locus and weaker associations of class I HLA variants, which are strongly implicated in type 1 diabetes heritability. A conditional analysis confirmed this weak association, highlighting a potential genetic discriminator between LADA and type 1 diabetes [54]. In twin studies the heritability of type 1 diabetes (and hence measurable genetic risk) decreases with age, yet still contributes a greater risk than is the case with many common complex diseases [61]. It is possible that, in future, with larger studies focused on adult type 1 diabetes, we may be able to better define genetic similarities and differences in type 1 diabetes across all ages. Currently, even with the limitation that most type 1 diabetes studies are carried out in children, it is likely that type 1 diabetes GRSs will be used in combination with other biomarkers, such as autoantibodies, to better identify and classify type 1 diabetes in individuals with features of both type 1 diabetes and other types of diabetes [45, 71].

## Future of genomics and genetics in healthcare

### GRS utility in population screening

Population screening is increasingly important to identify high-risk individuals with stage 1 and stage 2 type 1 diabetes, particularly as disease-modifying therapy is translated to clinical care [11]. As ~90% of individuals who develop type 1 diabetes do not have a relative with the disease, identifying individuals who will progress to stage 3 is challenging [50, 78]. Variation in incidence of new islet autoimmunity means that there is no single age at which autoantibodies will be detected in everyone who will progress to stage 3. It is possible, but potentially expensive, to implement screening at multiple time points. As genetic risk does not change with time, including a type 1 diabetes GRS as part of newborn screening could identify these individuals at highest risk for more intense monitoring, with autoantibody testing implemented in the minority of individuals in the population who account for the majority of type 1 diabetes cases [11]. Using GRS2 in simulation studies, Sharp et al showed that >77% of future type 1 diabetes cases can be identified within 10% of the general population, identifying a subset of individuals who may benefit from follow-up antibody screening [6]. Bonifacio showed that children in TEDDY with *DR3/DR4-DQ8* or *DR4-DQ8/DR4-DQ8* and a GRS >14.4 had a 11.0% risk of developing multiple autoantibody status by age 6 years compared with a 4.1% risk in children with a GRS ≤14.4 [59]. In the Global Platform for the Prevention of Autoimmune Diabetes (GPPAD) study [79], participants with a risk of multiple autoantibody development of >10%, informed by a GRS, received exposure to insulin prior to islet autoantibody development.

Population-based screening approaches for type 1 diabetes, using islet autoantibody status alone or combined with a GRS, have been further described by Sims et al [11].

### Combined risk scores and type 1 diabetes prediction

Generally, more accurate prediction models include multiple time-dependent clinical biomarkers [80]. The combination of multiple predictive factors, for example family history, autoantibody status and GRSs, may improve the prediction of type 1 diabetes through a combined risk score (CRS) [80]. A CRS in high-risk children aged 2 years (*n*=7798) significantly enhanced the prediction of type 1 diabetes (AUC≥ 0.92) compared with the use of autoantibody status alone [80]. A 30 SNP GRS combined with age, autoantibody status and Diabetes Prevention Trial-Type 1 risk score predicted progression to type 1 diabetes in at-risk family members (initially without diabetes) of TrialNet Pathway to Prevention participants (time-dependent AUC=0.73–0.79) [61]. Using genetics in CRSs is likely to help risk stratify individuals (initially without a diabetes diagnosis) who are either autoantibody negative or single autoantibody positive, alongside other risk scores that incorporate metabolic measures for predicting stage 1 and stage 2 type 1 diabetes [80].

### Genomics revolution

The increase in genetic discoveries has driven the development of GRSs. SNP genotyping of individuals is becoming increasingly cheap (<15 US cents per SNP); GRS development costs are mainly related to blood sampling and the extraction of DNA [7]. Once performed, SNP genotyping information can be interpreted across an individual’s lifetime, reducing the need for costly repeat testing. As the discriminative power of most type 1 diabetes GRSs is attributed to nine SNPs, determining type 1 diabetes risk will become an increasingly cheap endeavour, using either cheap custom assays or genetic data in healthcare records [7].

The increasing availability of direct-to-consumer testing (e.g. 23andMe, www.23andme.com/en-gb/) means that individuals are increasingly gaining access to their own genetic information. There is potential for tests to integrate genetic information to generate GRSs with relatively low costs and easy accessibility, although there are associated risks with respect to direct consumer interpretation [81].

### Availability and barriers to translation

Limited guidelines and regulations exist for assessing the clinical readiness of GRSs; however, contrasting methodologies and applications make providing a definitive estimate of GRS predictive performance difficult [14, 81]. Furthermore, issues exist around GRS study designs and analyses, for example uncertain population substructures can drive inaccuracies in GRSs [81]. Study heterogeneity means that GRS models require regulation, to ensure that scores are accurate and robustly capable of informing clinical practice [14].

GRS interpretation within healthcare has not been fully explored [81]. There is significant potential for type 1 diabetes GRSs to identify high-risk individuals for follow-up care and treatment; however, how to communicate GRSs to people with type 1 diabetes and how GRSs should inform clinical judgement need further research prior to their implementation in clinical care [81]. The clinical implications of incorrect GRSs depend on disease severity, the effect of non-genetic risk factors and the impact of interventions.

If GRSs are to be integrated into clinical care, sufficient regulations are necessary to prevent genetic discrimination. A lack of GRS use in cohorts of non-European ancestry remains a major limitation to their translation to clinical care [6, 13, 14, 81]. Protective guidelines are essential for recruiting under-represented ethnicities to minimise genetic determinist views that would disproportionately impact those facing greater social inequalities [81].

## Conclusion

The increase in genetic studies and GWAS has enhanced the utility of type 1 diabetes GRS estimates owing to the greater understanding of disease heritability. Given that the genetic component is very pronounced in type 1 diabetes and has already been well characterised, it is likely to lead the way in the application of GRSs to diagnosing a complex trait. Improvements in HLA capture and the greater number of larger population-based cohorts have enabled the development of GRS models with the ability to discriminate between type 1 diabetes and other diabetes phenotypes. Type 1 diabetes GRSs, alone and in combination with other factors, also have utility for predicting autoantibody onset and thus identifying individuals for recruitment to population screening studies for earlier therapeutic intervention. Despite advancements in type 1 diabetes GRS models, there is a clear need for models based on non-European ancestries to fully capture the genetics of type 1 diabetes. The lack of training for healthcare professionals in the use of GRSs and lack of regulations on their use should be addressed before type 1 diabetes GRSs are translated into clinical practice. Once these issues have been addressed, type 1 diabetes GRSs have significant potential for identifying and informing treatment in individuals with type 1 diabetes.

## Supplementary Information

Below is the link to the electronic supplementary material.

Supplementary file1 (PPTX 589 KB)

## Abbreviations

CRS: Combined risk score
GRS: Genetic risk score
GWAS: Genome-wide association studies
LADA: Latent autoimmune diabetes of adults
PRS: Polygenic risk score
T1DGC: Type 1 Diabetes Genetics Consortium
TEDDY: The Environmental Determinants of Diabetes in the Young
VNTR: Variable number of tandem repeats
