# Supplementary material for: Utility of genetic risk scores in type 1 diabetes
Source: Diabetologia. 2023 Jul 13;66(9):1589–600. doi: 10.1007/s00125-023-05955-y (PMC10390619; doi:10.1007/s00125-023-05955-y)
Supplement: Supplementary file 1 — Supplementary file1 (PPTX 589 KB) [file 125_2023_5955_MOESM1_ESM.pptx]

## Slide 1
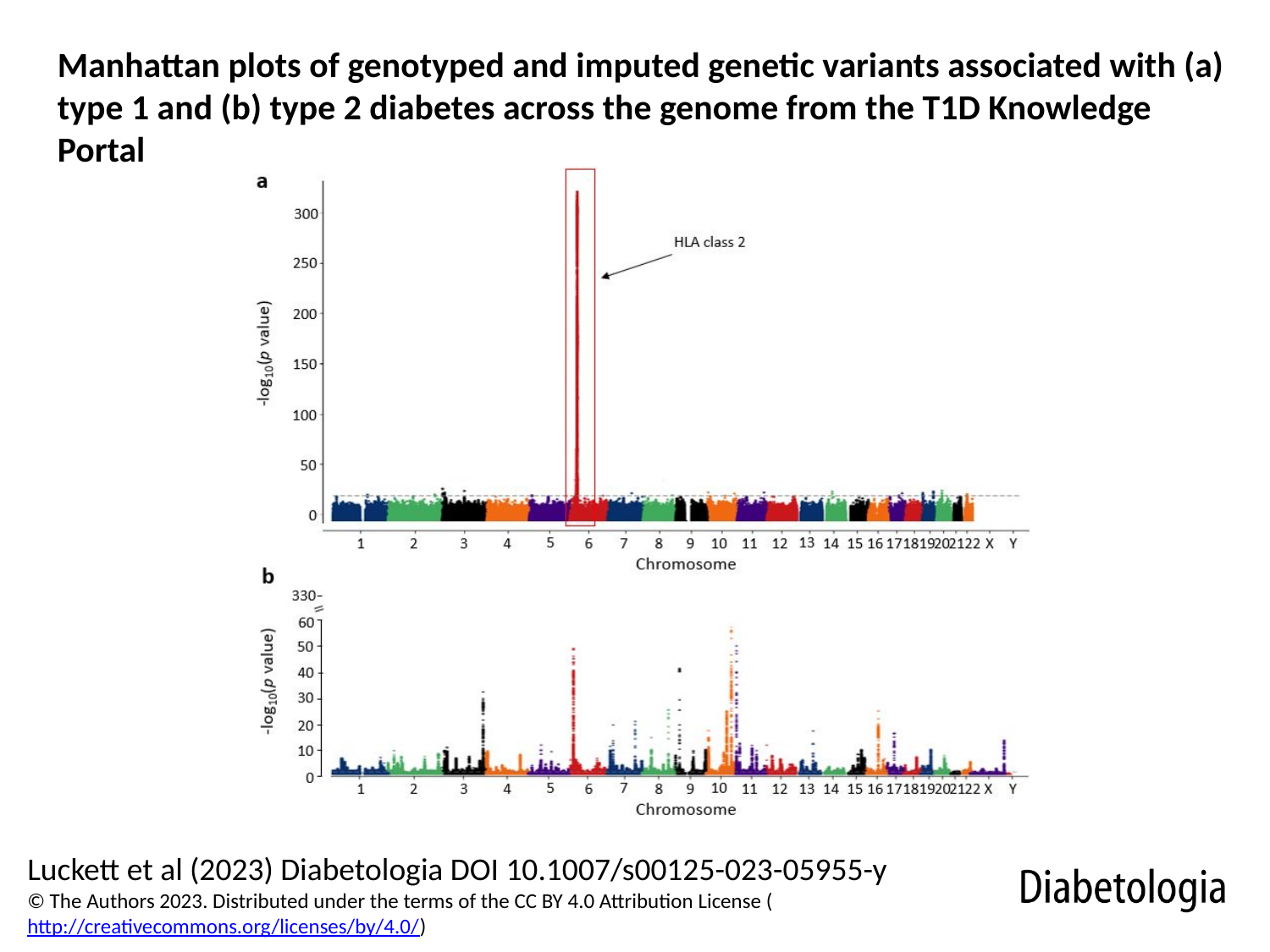

Manhattan plots of genotyped and imputed genetic variants associated with (a) type 1 and (b) type 2 diabetes across the genome from the T1D Knowledge Portal
Luckett et al (2023) Diabetologia DOI 10.1007/s00125-023-05955-y
© The Authors 2023. Distributed under the terms of the CC BY 4.0 Attribution License (http://creativecommons.org/licenses/by/4.0/)

## Slide 2
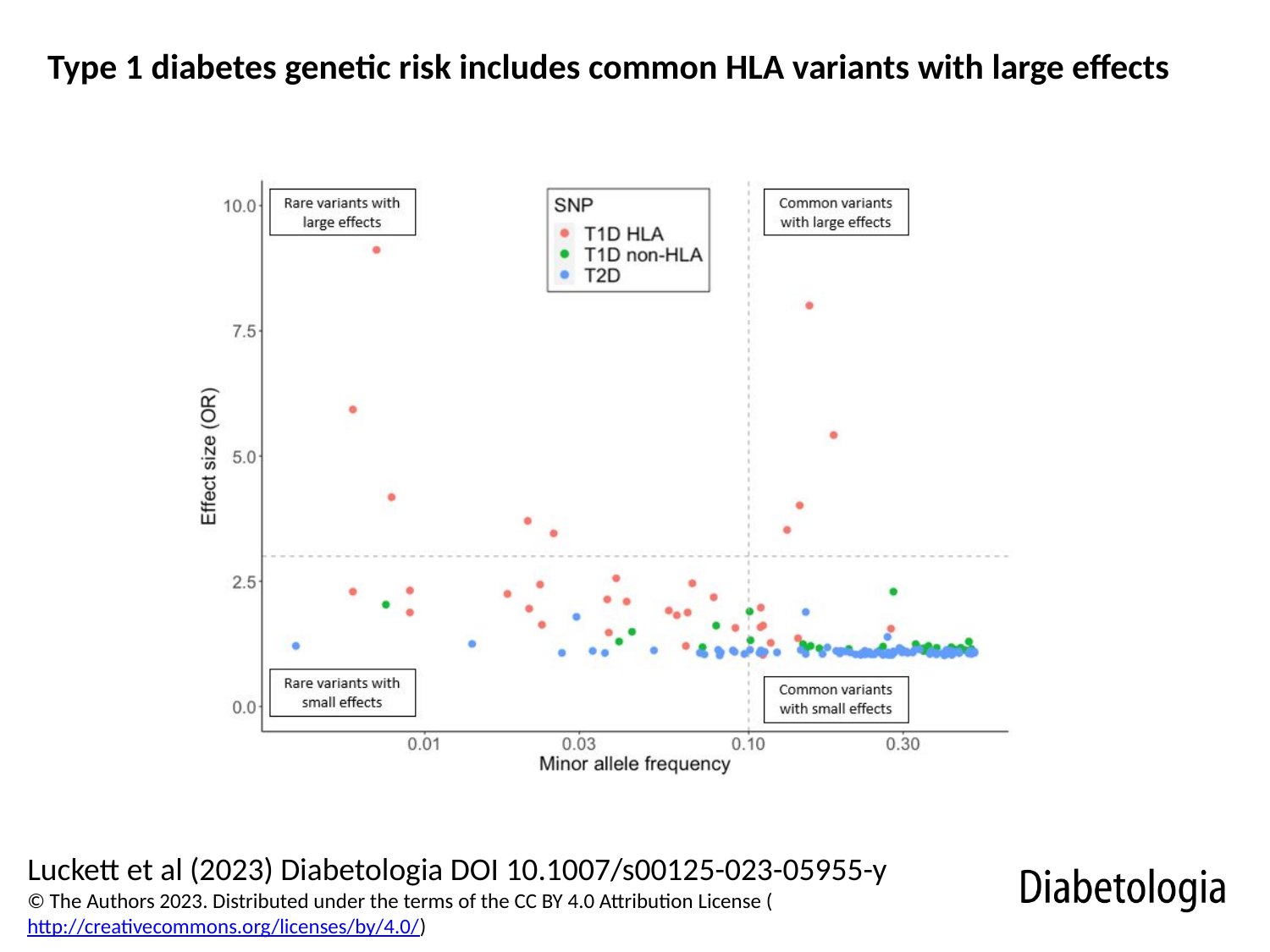

Type 1 diabetes genetic risk includes common HLA variants with large effects
Luckett et al (2023) Diabetologia DOI 10.1007/s00125-023-05955-y
© The Authors 2023. Distributed under the terms of the CC BY 4.0 Attribution License (http://creativecommons.org/licenses/by/4.0/)

## Slide 3
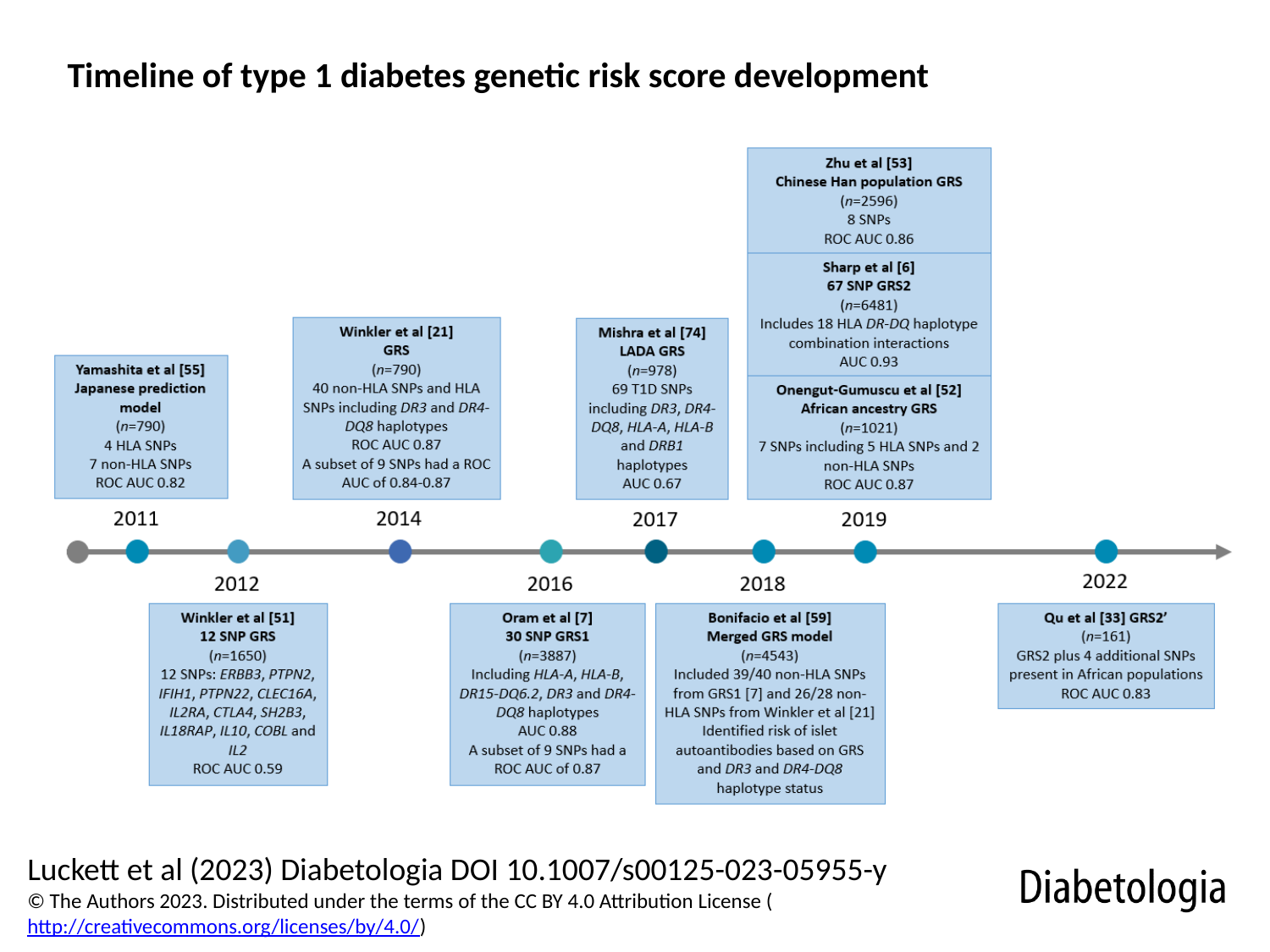

Timeline of type 1 diabetes genetic risk score development
Luckett et al (2023) Diabetologia DOI 10.1007/s00125-023-05955-y
© The Authors 2023. Distributed under the terms of the CC BY 4.0 Attribution License (http://creativecommons.org/licenses/by/4.0/)

## Slide 4
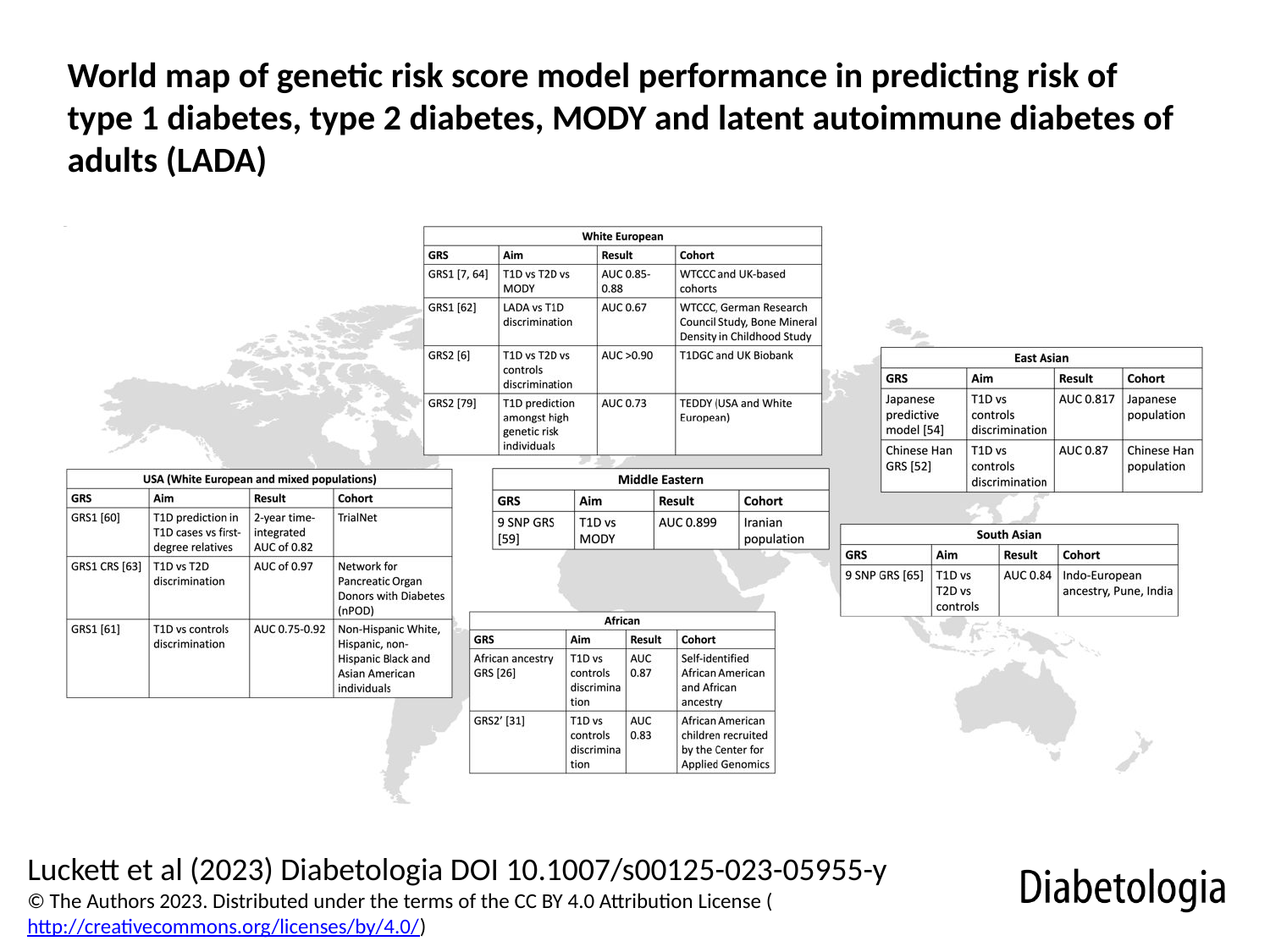

World map of genetic risk score model performance in predicting risk of type 1 diabetes, type 2 diabetes, MODY and latent autoimmune diabetes of adults (LADA)
Luckett et al (2023) Diabetologia DOI 10.1007/s00125-023-05955-y
© The Authors 2023. Distributed under the terms of the CC BY 4.0 Attribution License (http://creativecommons.org/licenses/by/4.0/)

## Slide 5
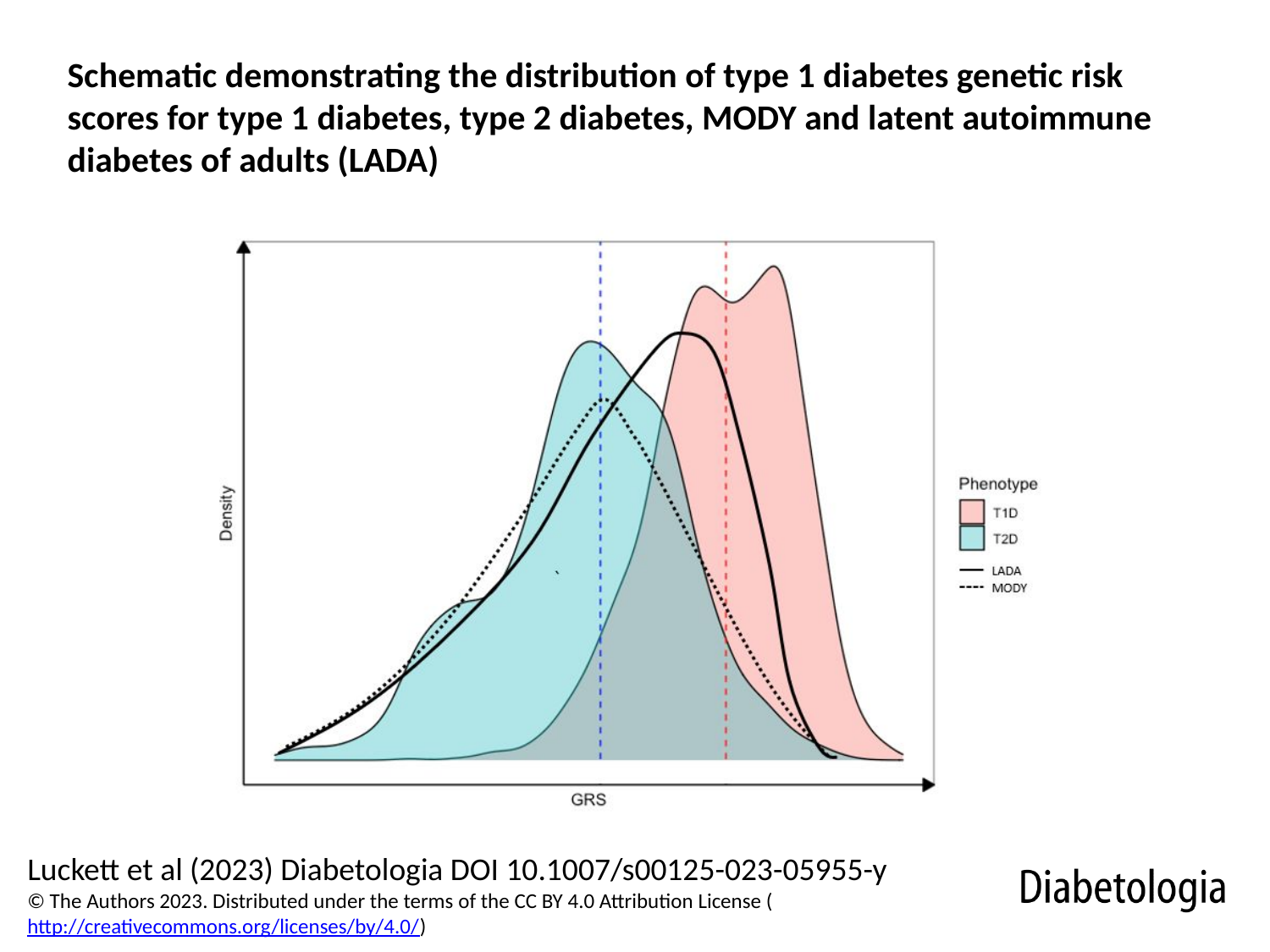

Schematic demonstrating the distribution of type 1 diabetes genetic risk scores for type 1 diabetes, type 2 diabetes, MODY and latent autoimmune diabetes of adults (LADA)
Luckett et al (2023) Diabetologia DOI 10.1007/s00125-023-05955-y
© The Authors 2023. Distributed under the terms of the CC BY 4.0 Attribution License (http://creativecommons.org/licenses/by/4.0/)
